# Disruption of Autolysis in *Bacillus subtilis* using TiO_2_ Nanoparticles

**DOI:** 10.1038/srep44308

**Published:** 2017-03-17

**Authors:** Eric McGivney, Linchen Han, Astrid Avellan, Jeanne VanBriesen, Kelvin B. Gregory

**Affiliations:** 1 Department of Civil and Environmental Engineering, Carnegie Mellon University, Pittsburgh, Pennsylvania, USA; 2 Center for the Environmental Implications of Nanotechnology (CEINT), Duke University, Durham, North Carolina, USA

## Abstract

In contrast to many nanotoxicity studies where nanoparticles (NPs) are observed to be toxic or reduce viable cells in a population of bacteria, we observed that increasing concentration of TiO_2_ NPs increased the cell survival of *Bacillus subtilis* in autolysis-inducing buffer by 0.5 to 5 orders of magnitude over an 8 hour exposure. Molecular investigations revealed that TiO_2_ NPs prevent or delay cell autolysis, an important survival and growth-regulating process in bacterial populations. Overall, the results suggest two potential mechanisms for the disruption of autolysis by TiO_2_ NPs in a concentration dependent manner: (*i*) directly, through TiO_2_ NP deposition on the cell wall, delaying the collapse of the protonmotive-force and preventing the onset of autolysis; and (*ii*) indirectly, through adsorption of autolysins on TiO_2_ NP, limiting the activity of released autolysins and preventing further lytic activity. Enhanced darkfield microscopy coupled to hyperspectral analysis was used to map TiO_2_ deposition on *B. subtilis* cell walls and released enzymes, supporting both mechanisms of autolysis interference. The disruption of autolysis in *B. subtilis* cultures by TiO_2_ NPs suggests the mechanisms and kinetics of cell death may be influenced by nano-scale metal oxide materials, which are abundant in natural systems.

Nutrient availability is often the critical factor limiting growth and controlling a bacteria population’s behavior^1^,^2^,^3^. In response to changing environmental conditions, bacteria participate in population-level behavior such as autolysis, cannibalism, and programmed cell death^3^,^4^,^5^,^6^,^7^,^8^,^9^. Autolysis, the self-digestion of the cell wall, is associated with several essential cell functions in bacteria, including spore formation, providing nutrients for persisting cells, and the transfer of genetic material to the remaining population^3^,^9^. Cannibalism is a precursor to sporulation in which non-sporulating sister cells have been shown to autolyse after toxins are produced by sporulating cells in the same population^5^,^6^. Autolysis and cannibalism have also been linked to biofilm maintenance and growth, and the secretion of lytic proteins^5^,^6^,^9^.

In gram-positive bacteria, such as *Bacillus subtilis*, the autolytic process occurs in the cell wall, which is comprised of a matrix of peptidoglycan, teichoic acids, autolysins, and a proton gradient. The aggregation of acidic proteins, such as teichoic acids, and electronegative peptidoglycan layers give rise to the negatively charged cell wall, where protons can accumulate as the proton motive-force (PMF). A balance of protons and counter ion binding sites associated with teichoic acids maintains the structural integrity and functionality of the cell wall matrix^10^,^11^. Jolliffe *et al*.^12^ demonstrated that when deprived of a carbon source, the PMF of *B. subtilis* collapses, leading to autolysis, characterized by the self-digestion of the cellular envelope and subsequent release of cytoplasmic materials to the surrounding environment (Fig. 1). It has been suggested that the released cellular matter may be used as substrate for the remaining population to persist^3^,^6^. Under more favorable cell growth conditions, where nutrients are abundant, autolysins are spatiotemporally activated within the peptidoglycan matrix in a controlled process where they mediate essential tasks such as peptidoglycan turnover, cellular division, and sporulation.

It has been estimated that 50% of wall-associated teichoic acids extend beyond the cell wall creating what could be imagined as “fluffy” region^13^,^14^,^15^, which would be the point of contact between a cell and a surface in the environment. While it is generally agreed upon that teichoic acids are responsible for limiting the activity of autolysins, there is no consensus on the mechanism of control. It has been proposed that the dispersal and subsequent protonation states of D-alanyl esters throughout the cell wall matrix could provide a unique network to systematically determine the activity of autolysins, which require unique ionic microenvironments^16^. Since the local environment determines the packing density of protons, and thus counter-ion binding sites, the activity of autolysins is concomitantly related^17^. A dissipation of the PMF results in a chain-reaction-like collapse where the orderly cell wall proton and charge distribution is lost, which could lead to the uncontrolled confirmation of teichoic acids, giving rise to unlimited autolytic activity.

The interaction between wall-associated teichoic acids and materials with high specific surface areas, such as nanoparticles, has been highlighted by Jiang *et al*.^18^, who demonstrated that metal oxide NPs (Al_2_O_3_, TiO_2_, and ZnO) adsorbed and altered the structure of cell wall biomolecules, including teichoic acids *in vitro*^18^. It was suggest that the resulting teichoic acid structural changes that occur when gram-positive bacterium and metal-oxide nanomaterials interact might be responsible for bacterial toxicity^18^. Through solid-state NMR studies, Wickman & Rice further concluded that, when lipoteichoic acids were simultaneously adhered to peptidoglycan, the positively charged alanine group binds to the surface of negatively charged TiO_2_^19^. This suggests that through their interactions, metal oxide NPs may influence cellular processes that are mediated through structural changes in cell-wall proteins, such as autolysis. However, to the best of our knowledge, no research has highlighted the influence of metal oxide surfaces on autolysis. We chose titanium dioxide nanoparticles (TiO_2_ NPs) as a model nanomaterial for its high production rate, consumer and industrial usage, and projected release into environmental compartments^20^,^21^,^22^. Furthermore, while TiO_2_ NPs are a semiconducting material that generate reactive oxygen species when irradiated with ultraviolet light^23^,^24^, in the absence of a UV source TiO_2_ NPs are not known to be particularly toxic to microorganisms^24^,^25^,^26^. Using *B. subtilis* as a model organism, the concentration dependence and kinetics of autolytic disruption, the impact of TiO_2_ NPs on the PMF and activity of released lytic enzymes, and the hyperspectral mapping of TiO_2_ deposition on the cell wall are described.

## Methods and Materials

### TiO_2_ nanoparticle preparation and characterization

Degussa P-25 TiO_2_ (70% anatase/30% rutile) NPs were suspended (0.3% wt.) in 15 mL of sterile lysis buffer (5 mM NaHCO_3_ buffer pH 7.0), *via* probe sonication (550 Sonic Disembrator, Fischer Scientific) with an acoustic power input of 4.11 W (determined using calorimetric method described by Taurozzi *et al*.^27^, see Supplementary Fig. S1) for 10 minutes in a glass beaker. To serve as a stock solution, the suspension was further diluted to 3 g/L and stored at room temperature in a glass bottle wrapped in aluminum foil. Prior to experimentation, the stock solution was diluted in the lysis buffer to 1000 ppm and probe sonicated (under the same power level as described above) for 10 min and then diluted to the desired TiO_2_ NP concentration.

Dynamic light scattering (DLS) measurements were collected on an ALV CGS-3 goniometer with ALV/LSE-5004 Light Scattering Electronics and Multiple Tau Digital Correlator. All samples were prepared in 5 mM NaHCO_3_ and adjusted to pH 7.7 using HCl or NaOH. Two-minute duration measurements were taken in triplicate on 1 mL of 10 ppm TiO_2_ NPs. Analysis of the autocorrelation function was performed using a constrained regularization algorithm in the ALV-7004 Correlator Software. Diffusion coefficients, *D*, were converted to hydrodynamic radius, *d*_*H*_ values using the Stokes-Einstein equation (1):


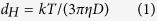


where *k* is the Boltzmann constant, *T* is the absolute temperature, and *η* is the viscosity of the solution. The electrophoretic mobility of TiO_2_ NPs was measured using a Malvern nano ZS Zetasizer (Conductivity: 0.557 mS.cm; Temperature: 24.9 °C; Count Rate: 172.8 kcps) and converted to zeta potential using the Smoluchowski relationship. The intensity-averaged hydrodynamic diameter of the TiO_2_ NPs, as determined by DLS, was centered around 250 nm (standard deviation: 8 nm) and the zeta potential was found to be −41.3 mV (standard deviation: 5 mV).

TEM imaging was used to observe the primary and aggregate dried TiO_2_ NPs size distribution. All TEM images were produced after *B. subtilis* cells were exposed to TiO_2_ NPs. TEM samples were fixed with 1% formaldehyde and 1% glutaraldehyde in 5 mM NaHCO_3_ for 24 hours at 4 °C. An aliquot of 1 μL was then placed on a carbon coated copper grid for examination. TEM images showed that TiO_2_ NPs were visible aggregates consisting of primary particles ~20 nm in diameter and distinct from cell components with higher electronic density (Supplementary Fig. S2). Some TiO_2_ NPs appeared to be closely associated with the cell wall of *B. subtilis*.

### Cell culture selection

*Bacillus subtilis* ATCC 6051 is a well-studied model organism for autolysis research^12^,^28^,^29^,^30^. It is ubiquitous in the environment and known to have several survival strategies, e.g., biofilm formation, spore formation, and motility^31^,^32^. It is a robust bacterium used in biotech applications, such as enzyme production^33^, and laboratory toxicity studies as a model gram-positive organism^34^.

### Preparation of cell cultures

Experimental cultures of *B. subtilis* were grown in 20 ml of LB broth-Miller (Fisher Scientific) at 37 °C, aerobically on a shaker plate, for 10 hours, to reach a concentration of ~10^8^ CFU/mL. Cells were harvested via centrifugation (4,000 × *g*, for 7 minutes at 4 °C) followed by resuspension of the pellet in lysis buffer to rapidly deplete the culture of a nutrient source. Washing was repeated twice.

### *B. subtilis*—nanoparticle exposures

The effect of TiO_2_ NPs on *B. subtilis* in a bicarbonate lysing buffer (5 mM NaHCO_3_, pH 7.7) was studied under five different NP exposure conditions: 0, 1, 10, 50, and 100 ppm. All trials were done in the dark to prevent reactive oxygen species generation. A volume of 1 mL washed *B. subtilis* culture (~10^8^ CFU/mL) was added to 9 mL of TiO_2_ NP suspension to reach desired TiO_2_ NP concentrations. The exposed *B. subtilis* were incubated at 25 °C in 20 mL glass culture tubes while being shaken. After 1, 2, 4, and 8 h of incubation, 0.3 mL of each suspension was removed for analysis. Viable cell concentrations were determined by plating and counting colony-forming units (CFU) on LB agar. Each treatment was performed in triplicate.

Subsequently, using the same conditions described above, TiO_2_ was added at three different time points after *B. subtilis* was washed and suspended in lysing buffer. First, 1 mL of washed *B. subtilis* culture (10^8^ CFU/mL) was added to three different reactors containing 9 mL of lysing buffer with no TiO_2_ NPs. Next, TiO_2_ NPs were added to the 3 independent culture tubes (final concentration of 50 ppm TiO_2_) at 0, 30, and 60 minutes, respectively. Aliquots of 0.3 mL were sampled and viable cell concentrations were determined via CFU plating as described above. Each treatment was performed in triplicate.

### Membrane potential (ΔΨ) and proton gradient (ΔpH)

The membrane potential (ΔΨ) and proton gradient (ΔpH) of *B. subtilis* cells was determined by the distribution of 3,3’-dipropylthiadicarbocyanine iodide (DiSC_3_(5)) between cells and the suspending medium as described earlier^35^. Due to the compensatory interaction between the transmembrane ΔpH and ΔΨ, a decrease in fluorescence is an indication of ΔpH dissipation^36^. The interaction between ΔΨ and ΔpH that governs the PMF is described by equation 2:


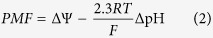


where *R* is the gas constant, *F* is the Faraday constant, and *T* is temperature.

An overnight culture of *B. subtilis* was grown and harvested as described above being resuspended in 30 mL of lysing buffer with either 0 or 50 ppm of TiO_2_ NPs. Immediately after the final resuspension, 3 mL of cell suspension was added to a quartz cuvette. 1 μL of 3 mM DiSC_3_(5) dissolved in DMSO was added to the cuvette, capped, and shaken by hand. The suspension was then monitored for fluorescence (Horiba Fluoromax-4 using FluorEssence software) at excitation wavelength 643 nm, slit width 4 nm, and emission wavelength 666 nm, slit width 4 nm. The fluorescence was monitored for 1 hour at intervals of 0.5 seconds with an integration time of 0.1 seconds. The emission signal from the photomultiplier tube was corrected by dividing by the reference signal from photodiode detector, which measures the output of the xenon lamp. Counts were kept below 2 million counts per second, as the detector is not linear over this count rate (Personal communication with Horiba technicians).

### Hyperspectral microscopy

The NP interactions with *B. subtilis* were assessed using an enhanced resolution dark–field microscope system (BX51, Olympus, USA) equipped with CytoViva Hyperspectral Imaging System (HSI, Auburn, AL). 20 μL of sample was deposited on a clean glass slide and covered with a coverslip for imaging. Hyperspectral images were acquired using 100% light source intensity and 0.6 s acquisition time per line. Each pixel of the hyperspectral image contains a light reflectance spectrum, ranging from 400 to 1000 nm with a spectral step of 1.5 nm. Each pixel thus has a spectral signature modulated by the nature of the material it contains^37^.

A spectral pre-library of TiO_2_ NPs was build using hyperspectral images of TiO_2_ NPs in lysing buffer (100 ppm). The pixels comprising endmembers hyperspectral signal were identified and grouped into the *TiO*_*2*_ *NPs pre-library*, following steps previously described by Badireddy and collaborators^37^. The specificity of the pre-library was assessed by mapping it on negative control images (abiotic lysing buffer and non-exposed cells in lysing buffer). Spectra in the pre-library that matched spectra of pixels in the negative control hyperspectral images were considered as unspecific false positives and removed from the pre-library. The remaining spectra built the final *TiO*_*2*_ *NP library*, containing specific hyperspectral TiO_2_ NP signature (see Supplementary Fig. S3 for specificity tests). This NP library was mapped on hyperspectral images (*B. subtilis* exposed to 0, 1 or 100 ppm during 1 hour in lysing buffer as described in the “Preparation of cell cultures” section above) to assess NPs-cells interactions. The mappings of the NPs pre-library and library was processed using a Spectral Angular Mapping algorithm (SAM, ENVI 5.2 software, in short, an algorithm comparing angles between vectors), whereby two vectors (*i.e*. spectra bands) with angles ≤0.09 rad were considered as similar. Pixels containing the NPs spectral signature were labeled with a chosen color of red. This method has already been tested and validated for bacteria-NP interaction^38^.

### Preparation of autolytic cell wall extracts

Cell wall associated autolysins and associated enzymes were extracted according to the method developed by Brown^39^. Briefly, *B. subtilis* strain was inoculated and maintained in LB Miller medium overnight before 2 liters of fresh LB medium was added and incubated for 12 hours with aeration by sterile air until the cell concentration reached 10^8^ CFU/ml. The cell suspension was further concentrated by centrifuging at 6000 × *g* for 10 min to a final concentration of 5% (wet weight) in 0.75% NaCl. The concentrated cell suspension was centrifuged once more at 6000 × *g* for 10 min. The supernatant was discarded and the pellet was resuspended in 2 L of 2 M LiCl solution. After one-hour of incubation at 4 °C, the suspension was centrifuged at 16,000 g for 15 min and the pellet was discarded. The cell-associated autolysin extracts were resuspended in the supernatant and stored at 4 °C for further experimentation.

### Adsorption of autolysins and peptidoglycan degradation

An autolysin extract isotherm on TiO_2_ NPs was measured by the solution depletion method, in which any protein concentration change, *ΔC*_*w*_, in the bulk solution prior to and after exposure to TiO_2_ NPs was attributed to adsorption to the surface, according to the following equation: *Γ* = *ΔC*_*w*_*V/A*, where *Γ* is the mass of protein adsorbed per surface area unit, *V* is the volume of the bulk solution, and *A* is the TiO_2_ NP surface area available for adsorption, calculated using the hydrodynamic radius and assumed spherical shape^40^.

1 mL Reactors were prepared by adding varying concentrations of TiO_2_ NPs in the range of 0–10,000 ppm to 55 mg/L of autolysin extract in lysing buffer. The suspensions were immediately capped and rotated for 24 h in the dark at 25 °C. The samples were then centrifuged at 16,000 × *g* for 15 min (Eppendorf Microcentrifuge 5415 D) to remove TiO_2_ NPs and any TiO_2_-adsorbed protein from the bulk solution. A portion of the supernatant was pipetted off and analyzed for protein concentration using the Pierce BCA assay Kit with BSA as the protein standard. The remaining portion of the supernatant (0.5 mL) was incubated with 1.5 mL of purified *B. subtilis* peptidoglycan (purchased from Sigma-Aldrich) to study the digestion process of peptidoglycan. The change in peptidoglycan concentration was monitored by measuring absorbance at 450 nm over time via a Cary 300 Bio UV-Visible Spectrometer/Cary WinUV software at room temperature.

### DNA and L-alanine concentration

Extracellular double-stranded DNA (dsDNA) was monitored using a PicoGreen dsDNA Kit (Molecular Probes, Invitrogen) coupled with a fluorometer (VersaFluor Fluorometer, Bio-Rad) following the supplier’s protocol. Two *B. subtilis* growth conditions were used to create standard curves: a positive control using liquid minimal Davis (MD) medium, and a negative control using lysing buffer. Cells were grown in MD medium because of its ability to keep *B. subtilis* cells vegetative. 0.2–0.3 mL samples were taken from each reactor at 0, 1, 2, 4, 8, and 12 hrs. Each sample point was diluted, with a portion being plated for CFUs, and 0.1 mL was used to determine dsDNA concentration. Cell suspensions were diluted to within the detection concentration of the PicoGreen dsDNA Kit (25 pg to 1 μg of dsDNA/mL). 0.1 mL of the kit reagent was added to 0.1 mL diluted sample and incubated in a glass cuvette at 25 °C for 3–4 min before being placed in a fluorescence reader with excitation at 480 nm and emission at 520 nm. L-alanine was monitored using an abcam L-Alanine Assay Kit (ab83394) according to the manufacturers protocol.

### Gene expression

To capture the influence of TiO_2_ NPs on *B. subtilis* gene expression, reverse transcription quantitative polymerase chain reaction (RT-qPCR) was used to detect RNA fold changes. Three genes were chosen for analysis: *skfA, sdpC*, and *lytC*; *rpoB* was used as an internal reference for normalization. Cells were grown, washed, and exposed as described in the “Preparation of cell cultures” section above. The washed cells were then exposed to either 100 ppm TiO_2_ NPs or no TiO_2_ NPs. Samples for RNA extraction were taken both right before exposure, *t* = 0, and one hour after exposure, *t* = 1 hr.

Total cellular RNA was extracted using a Qiagen RNeasy Mini Kit according to the manufacturer’s instructions. Purified RNA was verified by quantification using a Qubit 2.0 Fluorometer according to the Qubit RNA HS Assay Kit manufacturer protocol. Extracted RNA was then used as a template to synthesize first-strand complimentary DNA (cDNA) using an iScript cDNA Synthesis Kit according to the manufacturer’s instructions and using a BIO RAD MyCycler thermal cycler set at 25 °C for 5 minutes, 42 °C for 30 minutes, 85 °C for 5 minutes, and a holding temperature of 4 °C. Real-time PCR amplification of the cDNA was done using a Applied Biosystems 7500 Real Time PCR System. Primers used for PCR were designed using Integrated DNA Technologies PrimerQuest (http://www.idtdna.com/Primerquest) (Supplementary Table S1). Each 20 μL PCR mixture contained H_2_O (6.5 μL), forward primer (1 μL), reverse primer (1 μL), probe (0.5 μL), Taqman master mix (10 μL), and DNA sample (1 μL). The amplification program was set to 50 °C for 2 minutes (1 rep.), 95 °C for 10 minutes (1 rep.), 95 °C for 15 seconds (40 reps.), and 56 °C for 1 minute. The *rpoB* gene was used as a reference for data normalization. All the samples were analyzed in triplicate.

## Results and Discussion

Exposure of *B. subtilis* to TiO_2_ NPs in bicarbonate lysing buffer, under dark conditions, resulted in a negative correlation between NP concentration and loss of viable cell counts with time (Fig. 2). In the absence of NPs, the number of viable cells decreased by over 2.5 orders of magnitude in the first hour followed by 3 orders of magnitude loss over the next 7 hours (over a 5-log drop over the 8 hours monitored). A similar loss in cell viability was observed in the presence of 1 ppm of TiO_2_ NP. However, in the presence of 10 ppm TiO_2_ NPs, total viable cell counts decreased by less than 2 orders of magnitude over 8 hours. Viable cell counts further increased after exposure to TiO_2_ NPs at 50 and 100 ppm. The difference between 50 ppm and 100 ppm exposures is significant at the 8 hour time point and might be explained by a biphasic response common in toxicity response curves, where the 50 ppm range would be in the hormetic zone. Overall, exposure of *B. subtilis* to a nutrient-limited buffer of bicarbonate induced autolysis of cells, and that cell lysis was blocked by the presence of TiO_2_ NP in a dose-dependent manner.

**Figure 1:**
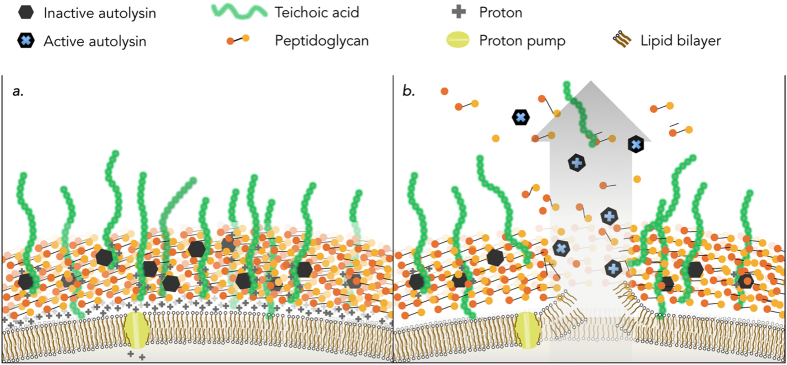
Schematic model and mechanisms of the cell wall and membrane of *Bacillus subtilis* (adapted from refs 10, 11, 12 and 16) under nutrient rich (**a**) and nutrient poor (**b**) conditions. (**a**) Under nutrient sufficient conditions, a cell is able to maintain it’s protonmotive-force (PMF) which allows protons to associate with teichoic acids embedded in the cell wall that keep the autolysins in their inactive form. (**b**) Autolysis: When nutrients are limited and the cells metabolism is unable to maintain the PMF, teichoic acids undergo a conformational change leading to an activation of autolysins that degrade the surrounding peptidoglycan ultimately resulting in turgor pressure rupturing the cell membrane and the release of internal and cell wall associated enzymes.

**Figure 2:**
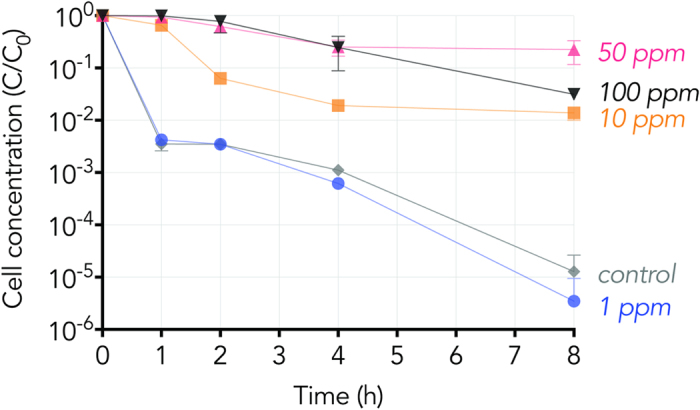
Colony Forming Unit (*C*) over time normalized to the initial Colony Forming Unit of each series (*C*_*0*_) of *B. subtilis* in 5 mM NaHCO_3_ buffer (pH 7.7) with exposure to 100 ppm (black upside-down triangle), 50 ppm (pink triangle), 10 ppm (orange square), 1 ppm TiO_2_ (blue circle), and a no TiO_2_ control (grey diamond) in the absence of light. Each point represents the mean (*n* = 3) with error bars representing the 95% confidence interval. The marker obscures error bars that appear absent. The lower error bounds are missing from two data points, *control* at 8 hours and *1* *ppm* at 8 hours, because the bottom of the error bar would go to a negative value which cannot be shown on a logarithmic axis. Connecting lines are provided to guide the eye.

**Figure 3:**
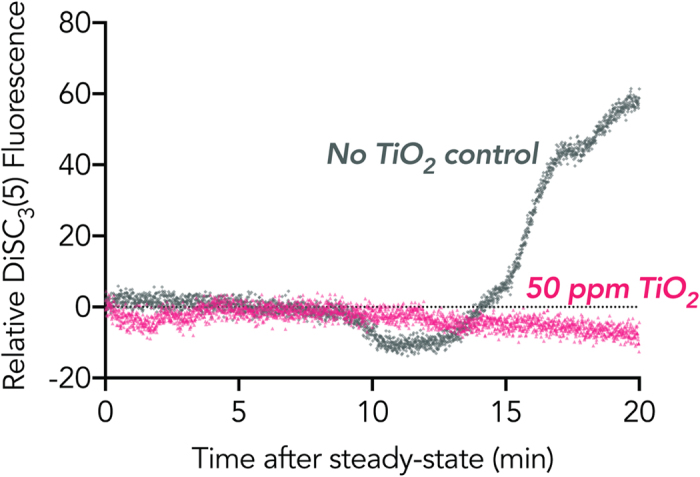
Fluorometery assay of DiSC3(5) stained *B. subtilis* cultures in 5 mM NaHCO_3_ in the presence of 50 ppm TiO_2_ (pink triangle) and absence of TiO_2_ (grey diamond) in the dark. Depolarization of *B. subtilis* membrane was monitored by measuring the fluorescence intensity at an excitation and emission wavelength of 643 nm and 666 nm, respectively, for 1 hour. Apparent equilibrium DiSC_3_(5) between the cell membrane and the media, as indicated by a flat-line in fluorescence, took 40 minutes.

The observation of cell lysis in nutrient-limited buffer agrees with previous findings that such solutions cause the onset of autolysis in *B. subtilis*^12^. Under nutrient poor conditions, a collapse of the protonmotive-force (PMF) in *B. subtilis* leads to a conformational change in associated teichoic acids and thus an activation of autolytic enzymes that digest the surrounding peptidoglycan (depicted in Fig. 1)^12^,^41^,^42^. Thus, the membrane potential (ΔΨ) and ΔpH of *B. subtilis* in lysing buffer was monitored using DiSC_3_(5) fluorescence. Apparent equilibration of the fluorescence signal between cells and the buffer, as indicated by a flat line in the fluorescence profile, took approximately 40 minutes. Ten minutes after fluorescence stability was reached (50 minutes after suspension in lysing buffer in the absence of TiO_2_ NPs), the fluorescence signal decreased before increasing 60 fold (Fig. 3). The slight decline and then sharp rise in fluorescence in the no TiO_2_ control indicates the collapse of the membrane potential. The dip in ΔΨ in the no TiO_2_ control is an indication of an increase in the membrane potential, which is likely the result of the dissipation of the membrane pH gradient due to the compensatory relationship between membrane pH and membrane potential (described in equation 2)^35^. When *B. subtilis* cells were suspended in lysing buffer in the presence of 50 ppm TiO_2_ NPs, minimal change in ΔΨ was observed over the same time period, indicating that the membrane potential, and pH gradient, remains intact. It is possible that the mode of prevention arises from the direct interaction between TiO_2_ NPs and the outer layer of wall teichoic acids that make up the bacteria-nanoparticle interface.

**Figure 4:**
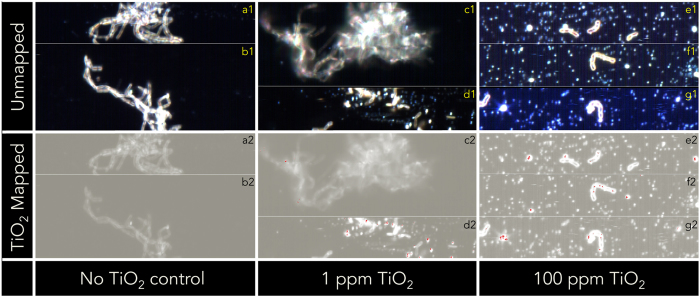
Unmapped (a1–g1) and TiO_2_ mapped (a2–g2) images of *B. subtilis* (~10^7^ cells/mL) exposed to 0 (**a,b**), 1 ppm (**c,d**), and 100 ppm (**e**–**g**) TiO_2_ nanoparticles suspended in bicarbonate buffer (5 mM). The TiO_2_ mapped images were obtained using Spectral Angle Mapper (0.09 rad), where all red pixels have spectral signatures identical to those in the TiO_2_ NP spectral library. Figures with the same alphabetical identifier, e.g. a1 and a2, are identical images, however, a2 is mapped to identify TiO_2_ location, whereas a1 is left unmapped.

Hyperspectral imaging (HSI) was performed on cultures of *B. subtilis* exposed TiO_2_ NPs to visualize TiO_2_ NPs—cell interactions (Fig. 4). HSI confirmed that at higher concentrations of TiO_2_ (100 ppm), the TiO_2_ spectral signature was consistently found to be associated with the surface of individual cells (Fig. 4e2,f2,g2) and bright dots we suspect to be TiO_2_ aggregates. Moreover, at 100 ppm TiO_2_ exposure, cells existed exclusively as individual, or short chained, planktonic cells (Fig. 4e1,f1,g1,f1,g1). These *in vivo* visualizations of TiO_2_ deposition on *B. subtilis* cell walls support previous *in vitro* findings which observed teichoic acid attachment to TiO_2_ sufaces^18^,^19^. At the experimental pH of 7.7, the surface of TiO_2_ is negatively charged (−41.3 mV), which would support electrostatic interactions between NP surface and positively charged alanine groups, as described previously^19^. It is possible that the protons on the surface of metal oxide NPs alter the microenvironment of the D-alanyl ester and teichoic acid interaction, affecting the activation of autolysin considering it has been demonstrated that the adsorption of a metal oxide surfaces influence the D-alanyl ester and teichoic acid interaction^18^. Therefore, a potential hypothesis for the disruption of autolysis by TiO_2_ NP is that association between NP surfaces and cell surface proteins gives rise to a conformational change in the surface proteins that regulate autolytic activity.

**Figure 5:**
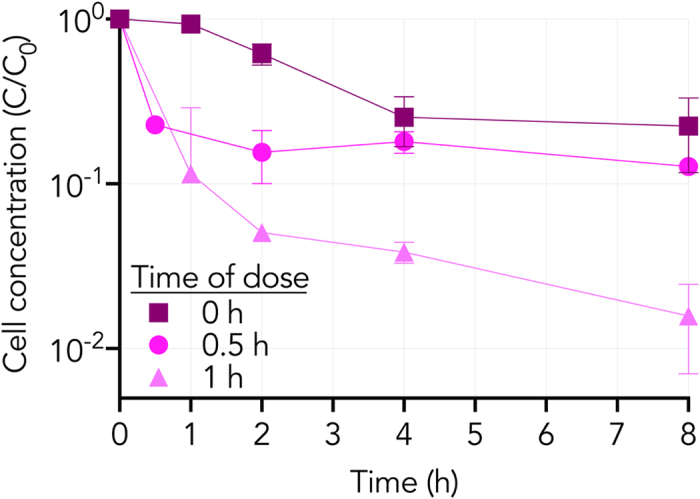
Colony Forming Unit (*C*) over time normalized to the initial Colony Forming Unit of each series (*C*_*0*_) of *B. subtilis* in 5 mM NaHCO_3_ buffer (pH 7.7) with the addition of 50 ppm TiO_2_ NPs at time points of 0 hour (square), 0.5 hour (circle), and 1 hour (triangle) in the absence of light. Each point represents the mean (*n* = 3 or 2) with error bars representing the standard deviation. The marker obscures error bars that appear absent. The lower error bound is missing from one data point: *t* = *1 hr* (triangle) at 1 hour because the bottom of the error bar would go to a negative value which cannot be shown on a logarithmic axis. Connecting lines are provided to guide the eye.

A kinetic assessment of cell lysis was performed in which TiO_2_ NPs were added before and after the apparent PMF collapse. 50 ppm of TiO_2_ NPs was dosed into cultures at 0, 30 and 60 minutes after the cells were suspended in lysing buffer (Fig. 5). Adding TiO_2_ NPs immediately (t = 0) after exposure of cells to lysing buffer led to a ~0.5-log decrease in cell counts after 8 hours. Adding TiO_2_ NPs 30 minutes after exposing cells to lysis buffer stabilized cell viability for the following 7.5 hours. When dosed at 60 minutes, after the observed PMF collapse, the following 7 hours saw a log drop in cell viability.

**Figure 6:**
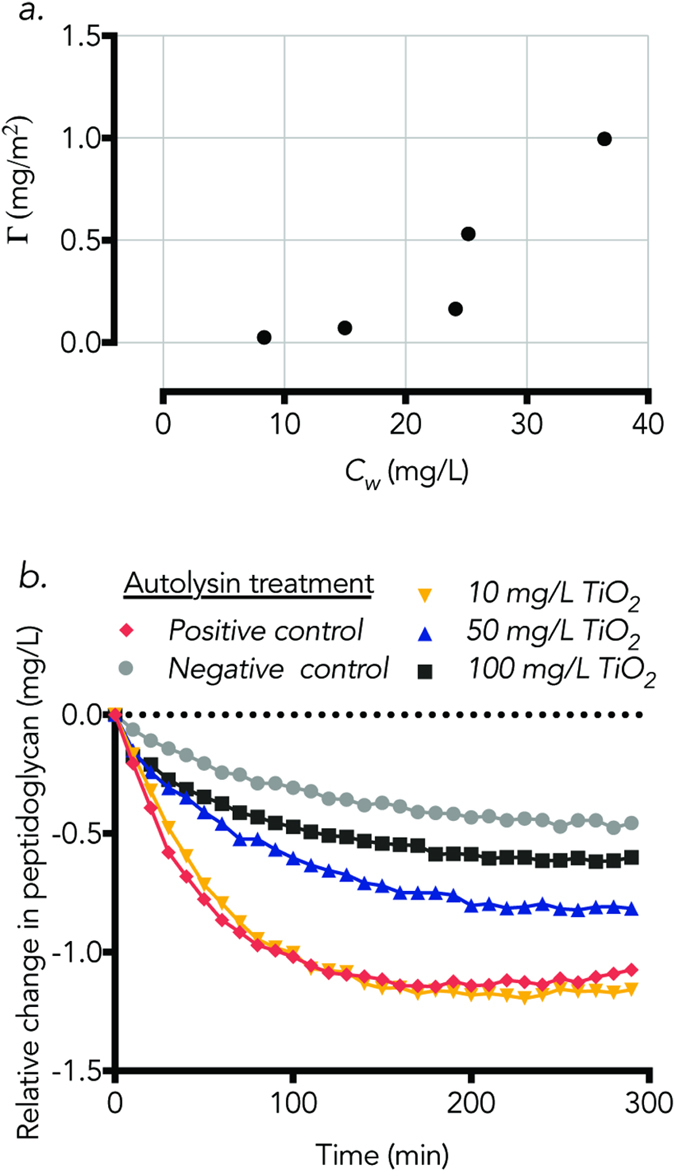
(**a**) Adsorption isotherm of cell wall enzyme extract on TiO_2_ NPs at pH 7.7 and 25 °C with the surface area concentration, *Γ*, plotted against the concentration of free enzyme in solution, *C*_*w*_. (**b**) Relative change in in peptidoglycan over time after incubation with cell wall associated enzymes. Prior to exposure to peptidoglycan, cell wall associated enzymes were incubated with varying concentrations of TiO_2_ NPs, which are denoted with distinct markers. The *Negative control* peptidoglycan was not exposed to any cell wall associated enzymes. The *Positive control* peptidoglycan was exposed to autolysins that were not incubated with TiO_2_ NPs. The dotted line represents the baseline of initial peptidoglycan concentration. Connecting lines are provided to guide the eye.

The observation that cell viability was maintained with higher efficiency when TiO_2_ was added before the observed PMF dissipation suggests that the release of active autolysins from cells that have already undergone autolysis may contribute to lysis of neighboring cells, and led us to consider another potential mechanism by which TiO_2_ NPs may interfere with a population undergoing autolysis: by adsorbing released autolysins from cells that have already lysed, limiting cell lysis proliferation. To test this, an adsorption isotherm was first produced for autolysins on TiO_2_ NPs (pH 7.7 and 25 °C, Fig. 6a). The autolysin extracts showed a binding affinity to TiO_2_ NPs, reaching a surface concentration of 1 mg/m^2^ (or 0.18 mg/mg TiO_2_). In a follow-up experiment to assess the functional activity of lytic enzymes after incubation with TiO_2_ NPs, autolysin extracts were exposed to varying concentrations of TiO_2_ NPs before being assayed for their ability to degrade peptidoglycan, the natural substrate of autolysins (Fig. 6b). Peptidoglycan degradation was inversely related to the concentration of TiO_2_ NPs present. After 5 hours, concentrations of peptidoglycan decreased by 29% and 57% when autolysins were incubated with 50 and 100 ppm TiO_2_ NPs, respectively. In the negative control (no autolysin), peptidoglycan decreased by 0.3 mg/L over 5 hours, whereas in the positive control (autolysins without TiO_2_ NP incubation), peptidoglycan was degraded by more than 1.2 mg/L over the same time period.

Generally, the degree of protein adsorption to NPs, and subsequent functionality, depends on both the NP surface and the protein’s folding structure, charge, and polydispersivity^43^. Protein adsorption to, and unfolding on, NPs can occur rapidly, within minutes of incubation^44^,^45^,^46^. The observed adsorption capacity of cell wall autolysin extracts to TiO_2_ NPs (Fig. 6a), and subsequent loss of peptidoglycan-degrading ability (Fig. 6b), could be contributed to the hydrophobicity of the autolysin extracts. It has been suggested that extracted autolysins may be linked to other membrane bound lipids of acids, implying hydrophobic properties^47^. Although protein structure analysis was not part of this work, and thus coming to a conclusion on the forces governing adsorption was not appropriate, the loss of functionality to digest peptidoglycan was correlated to the concentration of TiO_2_ incubated with the autolysins. These results are similar to a previous study by Xu *et al*.^48^ which demonstrated a change in structure and loss of functionality in lysozymes after incubation with TiO_2_ NPs^48^.

Supplementing the adsorption and peptidoglycan digestion assay results was the observation that the detectable TiO_2_ deposition at 1 ppm TiO_2_ exposure was associated with the large sticky aggregations of extracellular matter (Fig. 4c1–d2), where it is reasonable to believe starved cells are exuding general stress proteins and cytoplasmic material due to autolysis, leading to an accumulation of autolysins. At lower concentrations of NP exposure (1 ppm TiO_2_) there was no observable TiO_2_ deposition on cell walls (Fig. 4c2,d2). Of the very few observed individual planktonic cells in the 1 ppm TiO_2_ exposure, TiO_2_ was not mapped on any of them (contrary to HIS visualizations at 100 ppm TiO_2_).

Finally, as *B. subtilis* may form spores, it was also important to consider that the loss of viable cell counts could be due to cannibalism and spore formation, rather than, or in addition to, autolysis. Aliquots of *B. subtilis* cultures 12 hours after suspension in lysing buffer were removed and inoculated to fresh LB media where endospore germination could occur^49^. No growth was observed. *B. subtilis* are not known to form endospores when rapidly deprived of essential nutrients from the system^3^, as was done in this work. Additionally, spore formation takes 8 hours to complete^50^, at which time DNA is released by the mother cell. After 4 hours in the lysing buffer, the supernatant dsDNA concentration was over an order of magnitude higher than that in the maintenance medium (Supplemental Fig. S4a). An inverse relationship was observed between dsDNA concentration and CFUs. CFUs in the MD media had less than a 5% variation over the 12-hour monitored time period. In addition to dsDNA concentration, the release of L-alanine, one of the major components of peptidoglycan, was also monitored (Supplemental Fig. S4b). L-alanine is released into the extracellular environment when *B. subtilis* undergo autolysis^51^. L-alanine concentrations increased by a factor of three after 24 hours of suspension in lysing buffer. Cultures exposed to the lysing buffer were also examined in TEM micrographs and HSI; spores were not present. Furthermore, spore formation in *B. subtilis* is preceded by the expression of two main operons, sporulation killing factor (*skf*) and sporulation delay factor (*sdp*), in a process dubbed cannibalism^5^,^52^. Within these operons, there are two genes, *skfA* and *sdpC*, that are known to express lytic peptides that are responsible for lysing nonsporulating sister cells. The expression of these genes was monitored to determine if cannibalistic protein production was responsible for the loss of cell viability in cells suspended in lysing buffer, and to identify if TiO_2_ NPs had an influence on the genes expressed during cannibalism. After one hour of suspension in lysing buffer, there was less than a 1.5 fold increase of *skfA* and *sdpC* (Supplemental Figure S5). Furthermore, there was no significant difference in *skfA* or *sdpC* gene expression between cultures suspended in lysing buffer in the presence (50 ppm) or absence of TiO_2_ NPs. In addition to *skfA* and *sdpC*, the expression of *lytC* was also monitored. The *lytC* gene is responsible for producing a major autolysin, LytC, and is located throughout the vegetative cell wall^43^. As with the cannibalistic genes, there was no significant difference in *lytC* expression between *B. subtilis* suspended in lysing buffer in either the presence or absence of TiO_2_ NPs, revealing that TiO_2_ NP were not interfering with expression of autolytic genes. Based upon these analyses, complete loss of cell viability was attributed to autolysis.

## Conclusions

**Figure 7:**
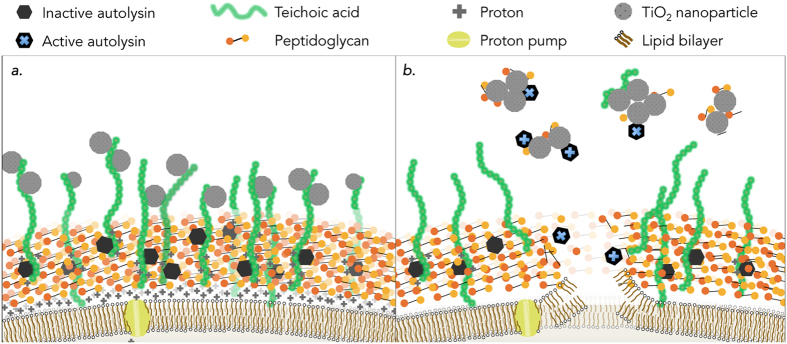
Proposed mechanisms through which TiO_2_ NPs influence autolysis in *B. subtilis*. (**a**) TiO_2_ NPs associate with the region of cell wall teichoic acids that make up the point of contact between the cell and NPs, altering autolysin activity and maintaining the PMF under nutrient limited conditions. (**b**) Once a cell has undergone autolysis, the released autolysins are adsorbed by TiO_2_ NPs which diminishes the peptidoglycan-degrading functionality, limiting enzymatic attack of peptidoglycan of other population members.

Results show that exposure of *B. subtilis* to a nutrient-limited buffer of bicarbonate induced autolysis in the population. Exposure of nutrient limited cultures of *B. subtilis* to TiO_2_ NPs appeared to prevent the propagation of autolysis in the population and enabled the cultures to survive nutrient stress that otherwise results in decimation of population numbers. TiO_2_ NPs prevented, or at least delayed, the dissipation of the protonmotive-force. It is possible that the mode of prevention arises from the direct interaction between TiO_2_ NPs and the outer layer of wall teichoic acids that make up the bacteria-nanoparticle interface. Hyperspectral imaging supported this hypothesis by revealing that high concentrations of TiO_2_ NP (100 ppm) was correlated with a decrease in cell aggregation and an increase in TiO_2_ deposition on the cell wall. Additionally, results indicated that released autolysins might lose peptidoglycan-degrading functionality after adsorption to TiO_2_ NP surfaces. Overall, the results suggest two potential mechanisms for the disruption of autolysis and cell death by TiO_2_ NP in a concentration dependent manner: (*i*) directly, through TiO_2_ NP deposition on the cell wall, delaying the collapse of the PMF and preventing the onset of autolysis; and (*ii*) indirectly, through adsorption of autolysins on TiO_2_ NPs, limiting the activity of released autolysins and preventing further lytic activity. (Depicted in Fig. 7).

The disruption of autolysis in *B. subtilis* cultures by TiO_2_ NPs suggests the mechanisms and kinetics of cell death may be complicated by metal oxide surfaces, which are common in environmental compartments. The association of nanoparticles to *B. subtilis* cell-wall and autolytic enzymes may not be confined to metal oxides, but rather linked to the surface chemistry of the particle once released in the environment^53^. Therefore, a variety of materials and surfaces are likely to influence autolysis, especially in natural systems such as groundwater where surfaces are abundant. If metal-oxide NPs delay autolysis through cell-wall associated protein interactions and/or limit the proliferation of autolysins via adsorption, as suggested with the results of the present study, the potential for NP to interfere with other cell-wall mediated interactions and extracellular signaling molecules should be explored. For example, disruption of autolytic activity may have an impact on growth-phase activity as autolysins in *B. subtilis* are involved in more controlled processes such as cell-wall turnover, cellular division, sporulation, and biofilm formation^54^. The ability to fine-tune the interactions between cells and nanoparticles through coatings that alter the electrosteric and electrostatic properties of particles suggests that the interference of cellular process using engineered nanomaterials may also be fine-tuned^55^.

## Additional Information

**How to cite this article**: McGivney, E. *et al*. Disruption of Autolysis in *Bacillus subtilis* using TiO_2_ Nanoparticles. *Sci. Rep.* **7**, 44308; doi: 10.1038/srep44308 (2017).

**Publisher's note:** Springer Nature remains neutral with regard to jurisdictional claims in published maps and institutional affiliations.

## Supplementary Material

Supplementary Information
