# Supplementary material for: Disruption of Autolysis in Bacillus subtilis using TiO2 Nanoparticles
Source: Sci Rep. 2017 Mar 17;7:44308. doi: 10.1038/srep44308 (PMC5355886; doi:10.1038/srep44308)
Supplement: Supplementary Information [file srep44308-s1.pdf]

Supplementary information

Disruption of Autolysis in *Bacillus subtilis* using TiO_2 Nanoparticles

Eric McGivney[*1,2], Linchen Han[1], Astrid Avellan[1,2], Jeanne VanBriesen[1,2], Kelvin B. Gregory[1,2]

[1]Department of Civil and Environmental Engineering, Carnegie Mellon University, Pittsburgh, Pennsylvania, USA

[2]Center for the Environmental Implications of Nanotechnology (CEINT), Duke University, Durham, North Carolina, USA

[*]Corresponding author: emcg@cmu.edu

Supplementary Table S1 Gene specific primer and probe sequences used for reverse transcription quantitative polymerase chain reaction in this study.

Gene	Forward	Reverse	Probe
skfA	CGATTGTGAAAGCTGCTG	ACACGTGTCATAGCAATACT	/56-FAM/ATGGGCTGT/ZEN/TGG GCCTCGAA/3IABkFQ/
sdpC	AACGCAGTTGCATTACAAA	AGC AGC CGC TTC TAA AT	/56-FAM/TGC AGT TGT /ZEN/TAC TGC GGC AGC /3IABkFQ/
lytC	CAGCTACCCTGACTCTATTG	TAC GGG CTC CTG TAG ATA A	/56-FAM/CAG GAG CTA /ZEN/CAC TGG CAG CT/3IABkFQ/
rpoB	CCAAGGTACGTGCTACAA	AGT TCA CCA AGC TCC ATA G	/56-FAM/AGC GTC CGA /ZEN/TCG TAA GTG TCG G/3IABkFQ/

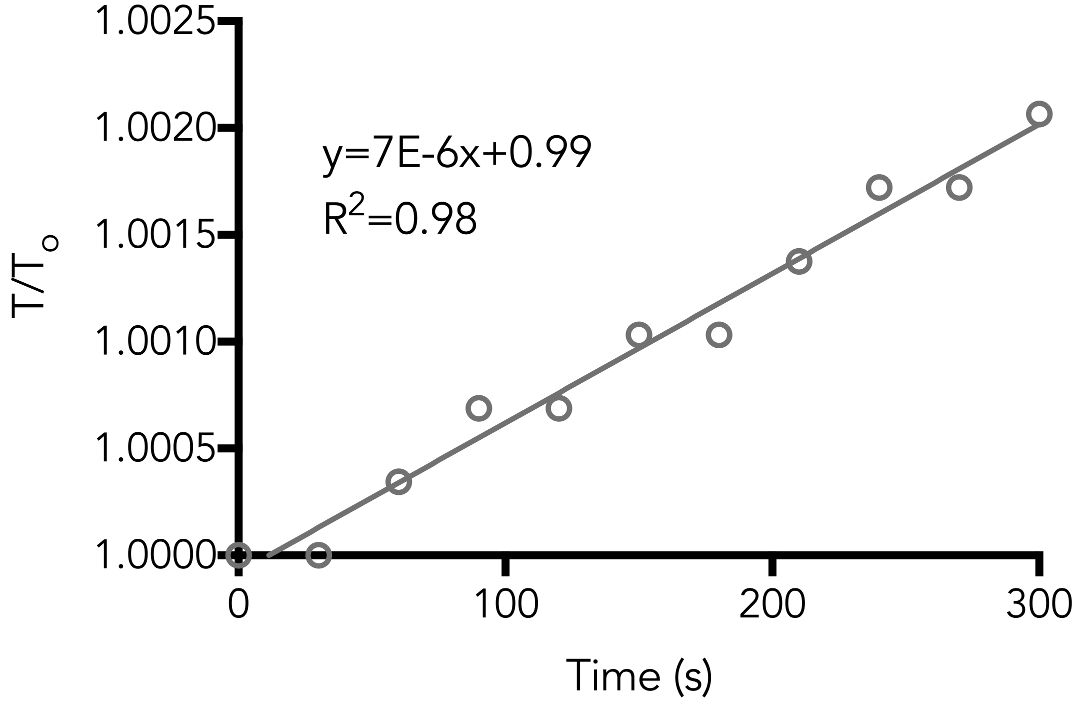

Supplementary Figure S1. Calorimetric curve, linear fit, and R^2 value of the fit. Recorded temperatures, T, are normalized to initial temperatures, T_o. The linear fit equation was used to calculate the delivered power of the sonicator, which was used to suspend TiO_2 nanoparticles into buffer.

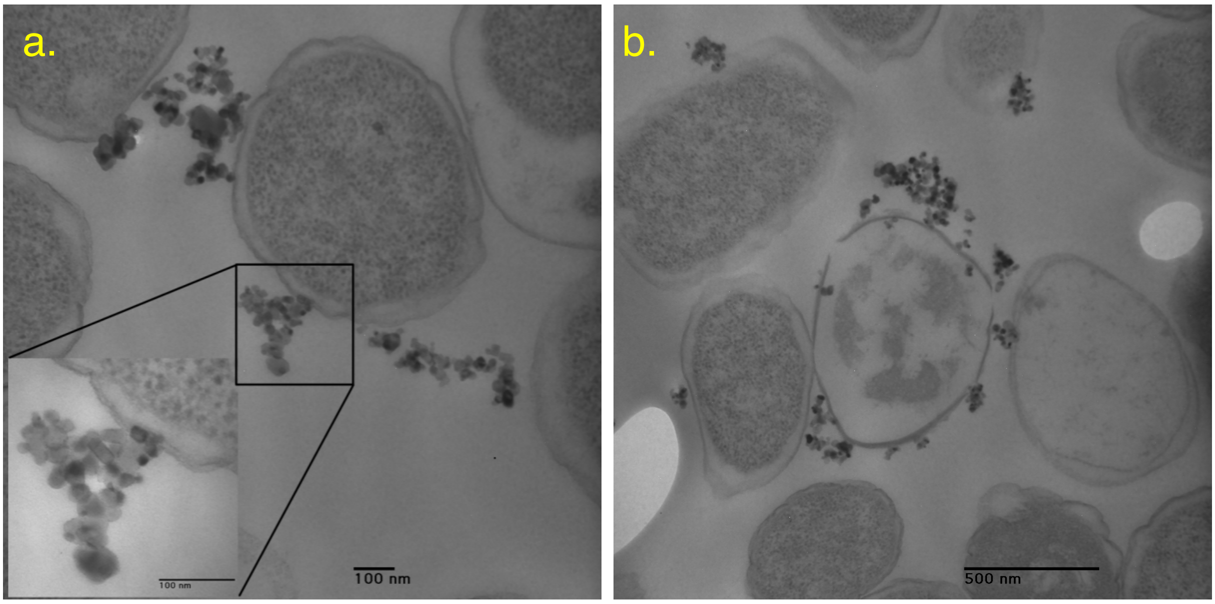

Supplementary Figure S2. TEM imaged of *B. subtilis* incubated with 50 mg/L TiO_2 NPs for 8 hour. (a.) Inset shows a close contact between TiO_2 NPs aggregates and cell wall. (b.) Some cells are lysing with lower electronic density (loss of intracellular substances).

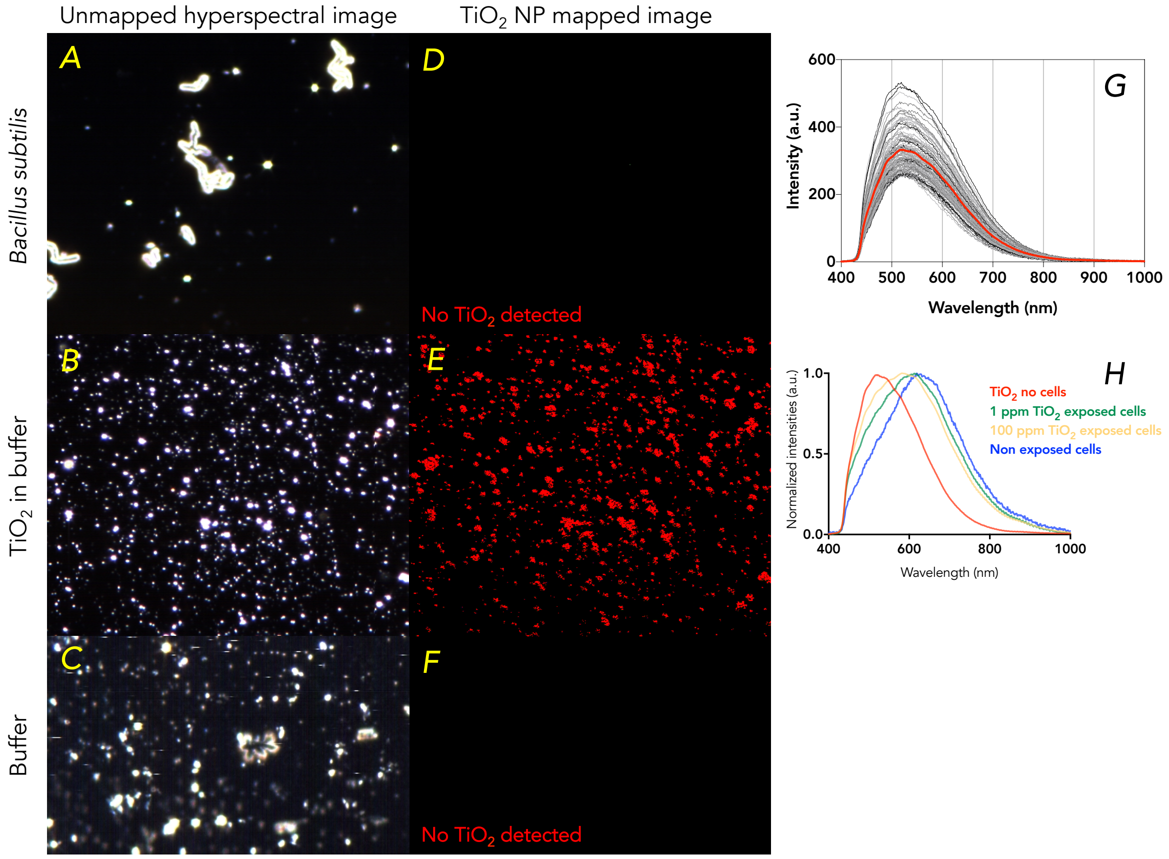

Supplementary Figure S3. Unmapped (A-C) and mapped (D-E) images of *B. subtilis* (~10^6 cells/mL), TiO_2 nanoparticles suspended (100 ppm) in buffer, and 5 mM bicarbonate buffer. The mapped images (D-F) were obtained using Spectral Angle Mapper (0.09 rad), where all red pixels have spectral signatures identical to those in the spectral library (G). The red profile in (G) highlights the average spectral profile of the TiO_2 NP library. (H) Shows a shift in cell wall spectral profile; as more TiO_2 is present in the sample, the more the cell wall spectral profile shifts to the left, towards the average spectral profile of the TiO_2 NP library.

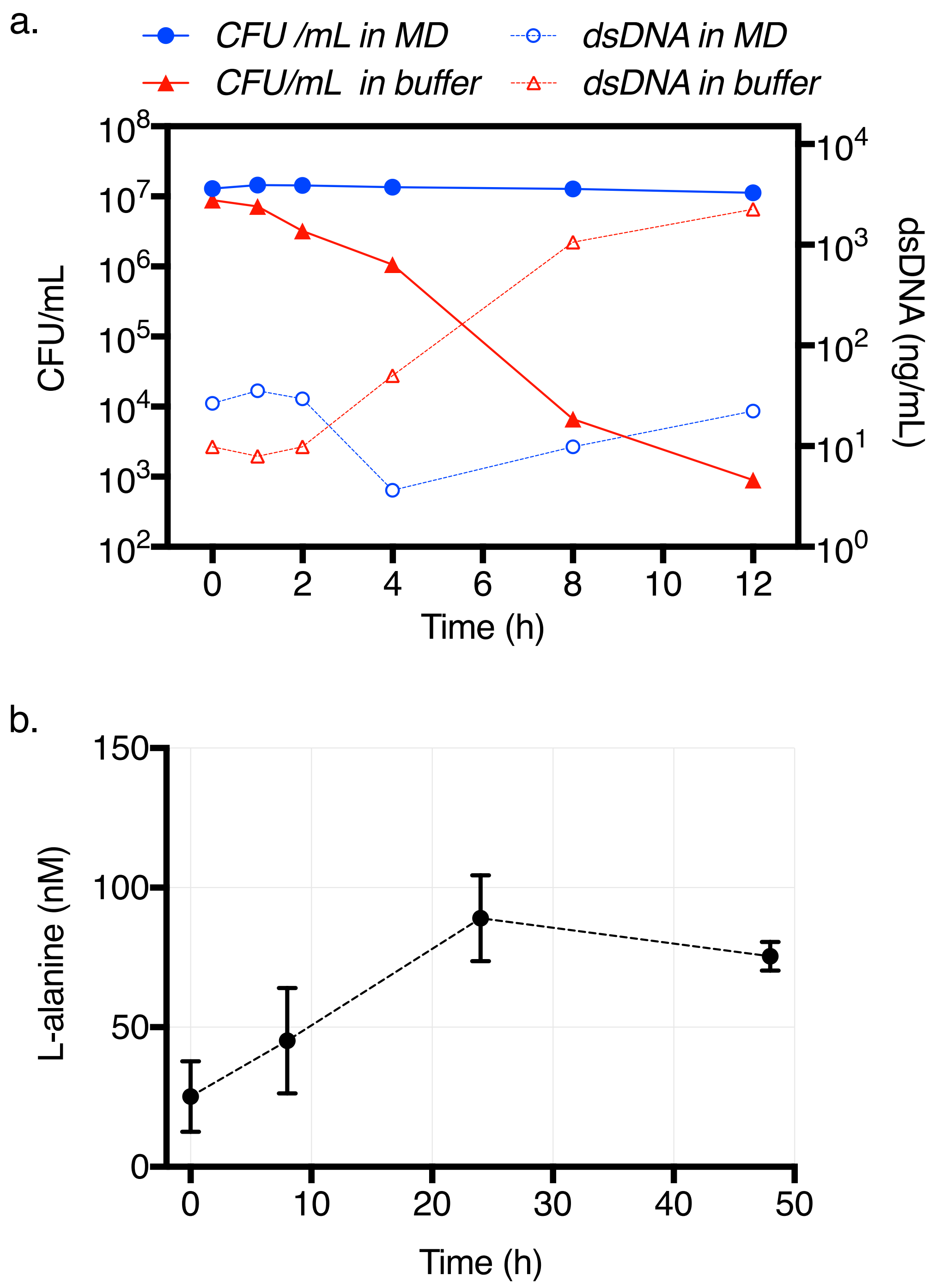

Supplementary Figure S4. (a.) Colony Forming Units per mL (CFU/mL, closed symbols) and double-stranded DNA (dsDNA, open symbols) of cultures of *B. subtilis* in minimal Davis media (blue) and 5 mM $NaHCO_3$ (red) over time. Connecting lines are provided to guide the eye. (b.) L-alanine concentration measured in the supernatant of *B. subtilis* suspended in 5 mM $NaHCO_3$. Each point represents the mean (*n*=3) and error bars represent standard deviation. Connecting line is shown simply to guide the eye.

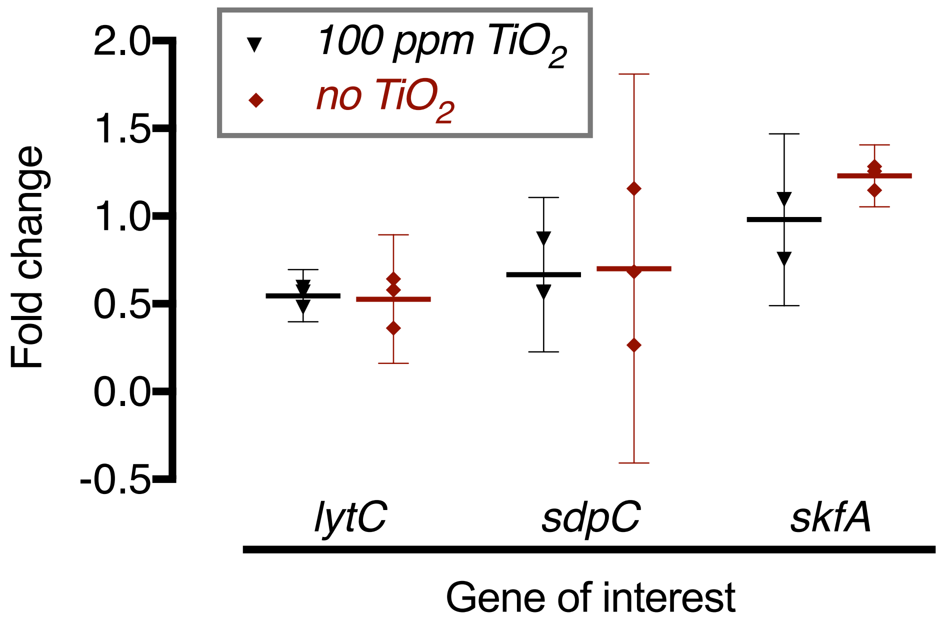

Supplementary Figure S5. RT-qPCR analysis of the expression of RNA encoding for *lytC*, *sdpC*, and *skfA* in cultures of *B. subtilis* after 1 hour of suspension in 5 mM $NaHCO_3$ in the presence (square) or absence (circle) of TiO_2 NPs presented as fold change relative to *rpoB*. Individual data points are plotted; thick bars represent the mean (*n*=3) and error bars represent the standard deviation.
